# Hsa_circ_0007456 regulates the natural killer cell-mediated cytotoxicity toward hepatocellular carcinoma via the miR-6852-3p/ICAM-1 axis

**DOI:** 10.1038/s41419-020-03334-8

**Published:** 2021-01-18

**Authors:** Min Shi, Zhao-Yun Li, Li-Ming Zhang, Xiao-Yu Wu, Shi-Hao Xiang, Yu-Gang Wang, Ya-Qiong Zhang

**Affiliations:** 1 Department of Gastroenterology, Tongren Hospital, Shanghai Jiao Tong University School of Medicine, Shanghai, China; 2 Department of Clinical Laboratory, Taizhou Central Hospital (Taizhou University Hospital), Taizhou, Zhejiang China

**Keywords:** Tumour biomarkers, Liver cancer

## Abstract

Circular RNAs (circRNAs) is one type of important non-coding RNAs that participate in tumorigenesis and cancer progression. In our previous study, we performed a microarray analysis of circRNAs between the tumor tissues and the adjacent normal tissues of hepatocellular carcinoma (HCC) patients, and found that the circRNA hsa_circ_0007456 is significantly downregulated in the tumor tissues and correlated with the prognosis of HCC. We further investigated the relationship between the expression levels of hsa_circ_0007456 in HCC and the susceptibility of NK cells, and found that the expression levels of hsa_circ_0007456 in HCC cell lines significantly influenced their susceptibility to NK cells. Through a series of screening and validation, we found that hsa_circ_0007456 mainly functioned through sponging miR-6852-3p and regulating the expression of intercellular adhesion molecule-1 (ICAM-1) in HCC. The miR-6852-3p/ICAM-1 axis is essential for the NK cytotoxicity toward HCC mediated by hsa_circ_0007456. In conclusion, we identify here hsa_circ_0007456 as a promising biomarker of HCC, and highlight hsa_circ_0007456/miR-6852-3p/ICAM-1 axis as an important signaling pathway in the process of tumor immune evasion and the tumorigenesis of HCC.

## Introduction

Hepatocellular carcinoma is the most common type of liver cancer and the fourth leading cause of cancer-related death all over the world^1^. The high risk factors of HCC include chronic hepatitis B and hepatitis C, alcohol addiction, metabolic liver disease and exposure to dietary toxins^1^. Emerging evidences indicate that the tumorigenesis of HCC is driven by multiple gene mutations (e.g. TERT, TP53, CTNNB1)^2,3^, and is participated by multiple factors within tumor microenvironment (TME)^4,5^. The tumor cells could also escape from the immune surveillance through a series of self-regulatory mechanisms, which still remain unclear to date^6,7^.

Circular RNAs, as one type of non-coding RNA (ncRNA), have been reported to participate in cancer occurrence and progression in a large number of studies^8,9^. CircRNAs can interact with microRNA or other molecules to regulate the gene expression in the transcript level^10,11^. In our previous study, we performed a microarray analysis of circRNAs between the tumor tissues and the adjacent normal tissues of HCC patients, and found that the circRNA hsa_circ_0007456 is significantly downregulated in the tumor tissues and correlated with the prognosis of HCC. However, the role of hsa_circ_0007456 in the tumorigenesis of HCC required more studies.

In this study, we investigated the relationship between the expression levels of hsa_circ_0007456 in HCC and the susceptibility of NK cells. Interestingly, we found that the expression levels of hsa_circ_0007456 in HCC cell lines significantly influenced their susceptibility to NK cells. Through a series of screening and validation, we found that hsa_circ_0007456 mainly functioned through sponging miR-6852-3p and regulating the expression of intercellular adhesion molecule-1 (ICAM-1) in HCC. The miR-6852-3p/ICAM-1 axis is essential for the NK cytotoxicity toward HCC mediated by hsa_circ_0007456. Altogether, this study provides novel insights into the functions of circRNAs in the process of tumor immune evasion and the tumorigenesis of HCC.

## Results

### The low expression of hsa_circ_0007456 is associated with the poor progression of HCC

**Fig. 1:**
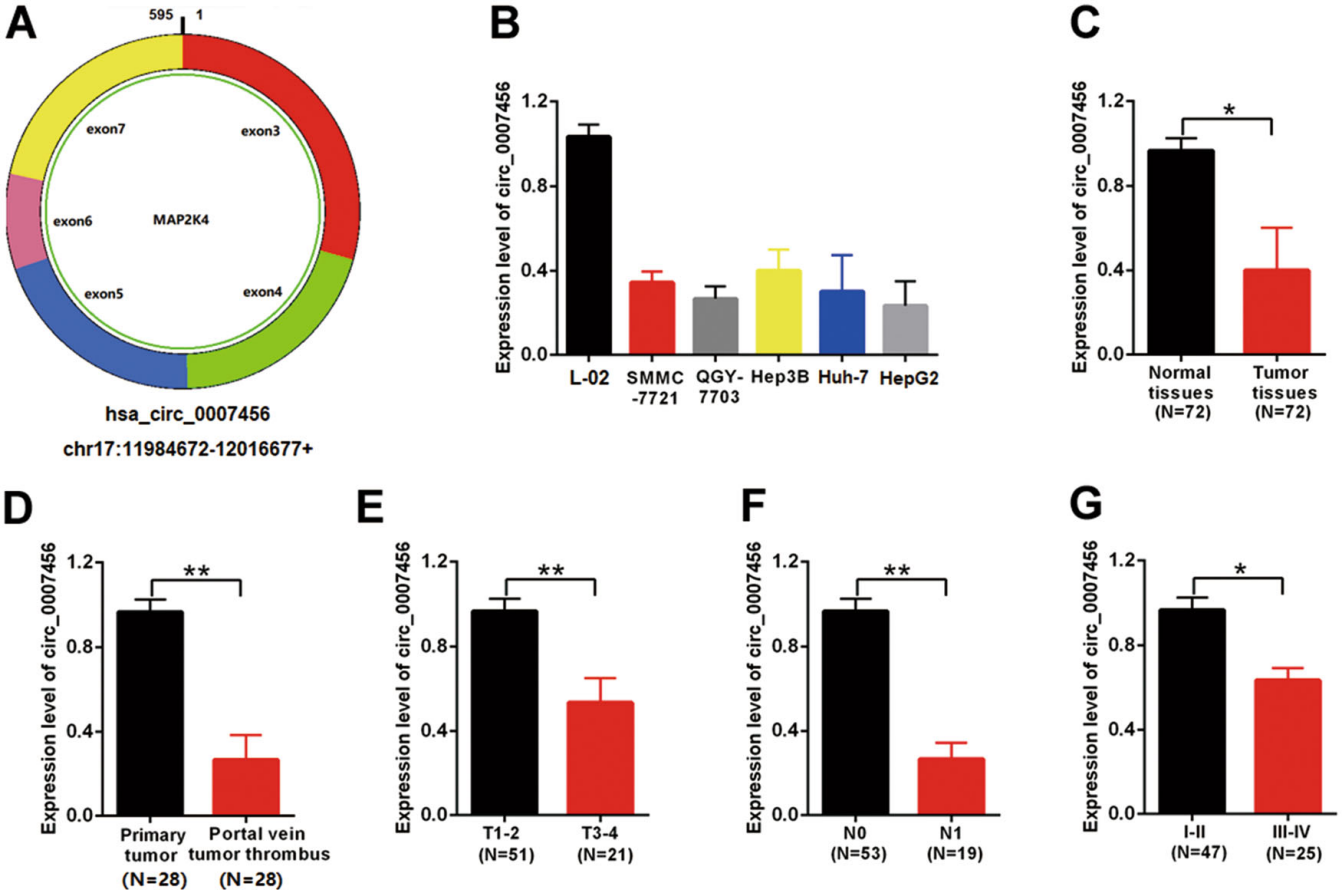
The expression of hsa_circ_0007456 is downregulated in HCC and is negatively correlated with the patients’ prognosis. **A** The schematic structure of hsa_circ_0007456. **B** The relative expression levels of hsa_circ_0007456 in the indicated cell lines. **C** The relative expression levels of hsa_circ_0007456 in tumor tissues and the adjacent normal tissues of the HCC patients (*n* = 72). **D** The relative expression levels of hsa_circ_0007456 in the primary tumor tissues (*n* = 28) and the portal vein tumor thrombus (*n* = 28). **E** The relative expression levels of hsa_circ_0007456 in the tumor tissues of T1-2 patients (*n* = 51) and T3-4 patients (*n* = 21). **F** The relative expression levels of hsa_circ_0007456 in the tumor tissues of N0 patients (*n* = 53) and N1 patients (*n* = 19). **G** The relative expression levels of hsa_circ_0007456 in the tumor tissues of stage I–II patients (*n* = 47) and stage III-IV patients (*n* = 25). Data are presented as mean ± SD. *p < 0.05; **p < 0.01.

Firstly, we examined the expression of hsa_circ_0007456 (Fig. 1A) in the HCC cell lines (SMMC-7721, QGY-7703, Hep3B, Huh-7, and HepG2) by RT-qPCR, and found that the expression levels of hsa_circ_0007456 in these cell lines were significantly lower than the immortalized human liver cell line L-02 (Fig. 1B).

Meanwhile, we collected the primary tumor tissues and the adjacent normal tissues from 72 HCC patients treated with liver resection at Taizhou Central Hospital Affiliated to Taizhou College between April 2015 and August 2017. Of the total 72 HCC patients, 53 are male and 19 are female (Table 1). There were 20 patients under 55 years old and 52 patients above at the time point of receiving surgery (Table 1). The evaluation of the primary tumor stages demonstrated that there were 51 cases in T1–T2 stages and 21 cases in T3–T4 stages (Table 1). The assessment of the lymph node metastasis demonstrated that there were 53 cases in N0 stage and 19 cases in N2 stage (Table 1). The detection of HBV infection demonstrated that there were 61 positive cases and 19 negative cases (Table 1). The genetic testing demonstrated that there were 41 cases with TP53 mutations and 31 cases with wild-type TP53 (Table 1).

**Table 1:** Relationship between circ_0007456 expression and the clinical pathological characteristics of HCC patients (*n* = 72).

Variable	No. of cases	circ_0007456 expression		*P* value
		Low expression	High expression	
*Age*				
≤55	20	10	10	*P* > 0.05
>55	52	28	24	
*Gender*				*P* > 0.05
Male	53	25	28	
Female	19	11	8	
*Primary tumor (T) stage*				
T_1_–T_2_	51	17	34	*P* < 0.05
T_3_–T_4_	21	15	6	
*Lymph node(N) metastasis*				
N_0_	53	17	36	*P* < 0.05
N_1_	19	12	7	
*HBV infection*				
Yes	61	30	31	*P* > 0.05
No	11	5	6	
*TP53 mutation*				
Yes	41	20	21	*P* > 0.05
No	31	16	15	

We firstly compared the expression levels of hsa_circ_0007456 in the primary tumor tissues and the adjacent normal tissues. Similar to the differences in cell lines, the expression levels of hsa_circ_0007456 in the tumor tissues were significantly lower than the normal tissues (*p* < 0.05, Fig. 1C). Then we asked whether the expression levels of hsa_circ_0007456 were associated with the progression of HCC. We analyzed the expression of hsa_circ_0007456 in the tumor samples from the patients with portal vein tumor thrombus (*n* = 25), and found that the expression levels of hsa_circ_0007456 in tumor thrombus were significantly lower than the primary tumor tissues (*p* < 0.01, Fig. 1D). Next, we classified the 72 patients into groups according to TNM staging system, and analyzed the expression of hsa_circ_0007456 in different groups (Fig. 1E–G). We found that the expression levels of hsa_circ_0007456 in tumor tissues of T3–4 group (*n* = 21), N1 group (*n* = 19) or III–IV group (*n* = 25) were significantly lower than T1–2 group (*n* = 51), N0 group (*n* = 53) or I–II group (*n* = 47), respectively (*p* < 0.01, *p* < 0.01, and *p* < 0.05 respectively, Fig. 1E–G).

To further analyze the relationship between the expression of hsa_circ_0007456 and the varying backgrounds of those HCC patients, we also tested the distribution of the high expression and the low expression of hsa_circ_0007456, which were separated by the median of expression levels, in the HCC patients with different clinical pathological characteristics with *χ*^2^ test (Table 1). Interestingly, we found that the expression of hsa_circ_0007456 was not significantly influenced by the age, the gender, the conditions of HBV infection or TP53 mutations (*p* > 0.05, Table 1), but was significantly associated with the primary stage and the status of lymph node metastasis (*p* < 0.05, Table 1). For the primary tumor stage, the ratio of low hsa_circ_0007456 expression in T3–T4 stage was significantly higher than that in T1–T2 stage (15/21 vs. 17/51, *p* < 0.05, Table 1). For the lymph node metastasis stage, the ratio of low hsa_circ_0007456 expression in N0 stage was significantly higher than that in N1 stage (12/19 vs. 17/53, *p* < 0.05, Table 1). These results suggest that hsa_circ_0007456 may function as a tumor suppressor in progression of HCC, especially the process of tumor metastasis.

### The expression levels of hsa_circ_0007456 in HCC cell lines could influence their susceptibility to NK cells

**Fig. 2:**
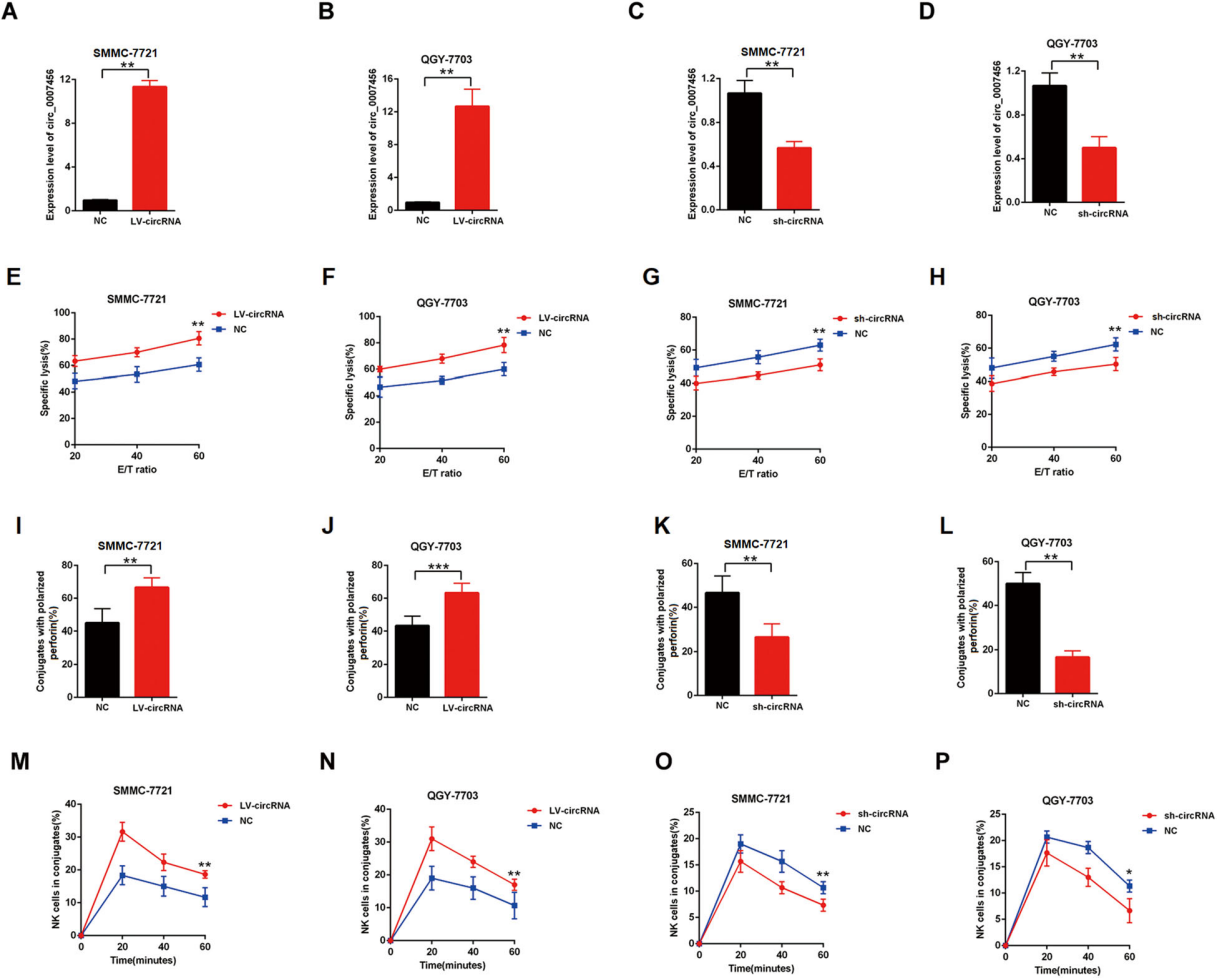
The expression levels of hsa_circ_0007456 in HCC were associated with their susceptibility to NK cells. After infection with hsa_circ_0007456-overexpressing or shRNA lentivirus or the control lentivirus in SMMC-7721 or QGY-7703: **A**–**D** The expression levels of hsa_circ_0007456 in the infected cells were determined by RT-qPCR; **E**–**H** The NK cell cytotoxicity to the infected cells was assessed by calcein release assays in indicated E/T ratios; **I**–**L** The numbers of perforin-containing NK cells against the infected cells were counted and analyzed; **M**–**P** The conjugate formation between NK cells with the infected cells was assessed by conjugation assay. Data are representative of three independent experiments and are presented as mean ± SD. *p < 0.05; **p < 0.01.

It has been known that NK cell is an antitumor innate immune effecter that can suppress the tumor metastasis^12–14^. We asked whether the expression levels of hsa_circ_0007456 in HCC influenced the sensitivity of NK cells. Thus, we constructed hsa_circ_0007456-overexpressing cell lines (Fig. 2A, B) and hsa_circ_0007456-downregulated cell lines (Fig. 2C, D), and examined their susceptibility to NK cells through the calcein release assay (Fig. 2E–H), perforin polarization assay (Fig. 2I–L) and conjugation assay (Fig. 2M–P). We found that the hsa_circ_0007456-overexpressing cells were more susceptible than the control cells (Fig. 2E, F, I, J, M, N). In contrast, the hsa_circ_0007456-downregulated cells were less susceptible than the control cells (Fig. 2G, H, K, L, O, P). These results suggest that the expression levels of hsa_circ_0007456 in these cells could indeed influence their susceptibility to NK cells.

### The hsa_circ_0007456 acts as a sponge of miR-6852-3p

**Fig. 3:**
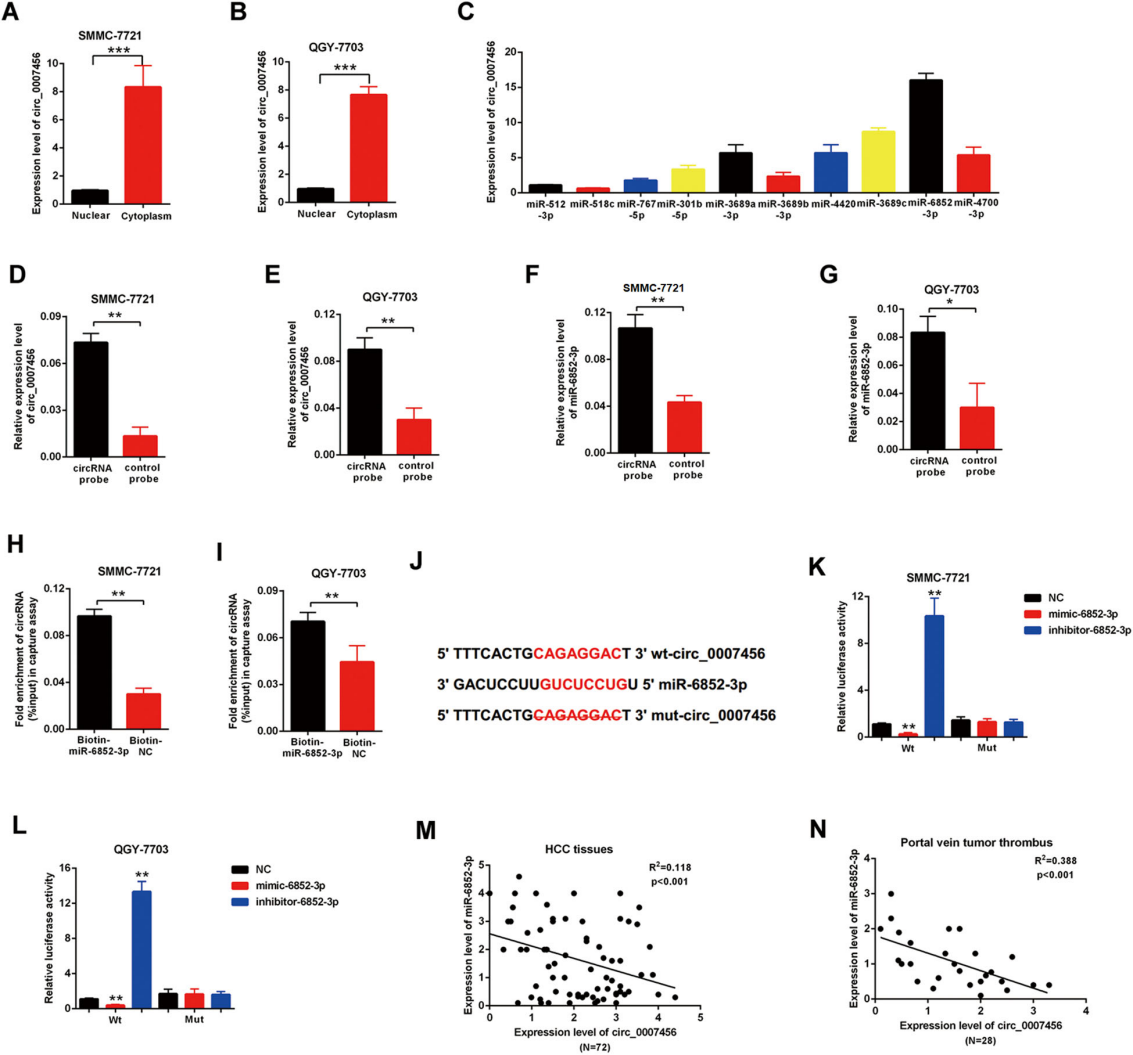
miR-6852-3p is the direct target of hsa_circ_0007456. **A**, **B** The relative expression levels of hsa_circ_0007456 in nuclear and cytoplasm of SMMCC-7721 (**A**) or QGY-7703 (**B**) were quantified by RT-qPCR. **C** The relative levels of hsa_circ_0007456 were quantified by RT-qPCR after pull-down with indicated microRNA probes. **D**–**G** After pull-down with hsa_circ_0007456 specific probe or the control probe in SMMCC-7721 or QGY-7703 cell lysis, the levels of hsa_circ_0007456 (**D**, **E**) and miR-6852-3p (**F**, **G**) were respectively quantified by RT-qPCR. **H**, **I** After pull-down biotin-coupled miR-6852-3p or the control probe in SMMCC-7721 or QGY-7703 cell lysis, the enrichment levels of the hsa_circ_0007456 were quantified by RT-qPCR. **J** The predicted binding motif between hsa_circ_0007456 and miR-6852-3p is indicated in red, and the mutant hsa_circ_0007456 was designed without the complementary sequence. **K**, **L** SMMCC-7721 or QGY-7703 stably expressing the luciferase construct containing the wild-type (WT) or mutant (MT) hsa_circ_0007456 were respectively transfected with miR-6852-3p mimic, inhibitor or the negative control (NC), and then the luciferase activity was examined. **M**, **N** The regression analysis of correlation between the expression of hsa_circ_0007456 and miR-6852-3p in HCC tissues (**M**) and portal vein tumor thrombus (**N**). Data are representative of three independent experiments and are presented as mean ± SD. *p < 0.05; **p < 0.01; ***p < 0.001.

The role of circular RNA as a sponge in regulation of microRNA has been well known^10^. In order to find out the potential target of hsa_circ_0007456, we used the Arraystar’s home-made miRNA target prediction software to scan globally the matching microRNA (Supplementary Table 1 and Table 2). To detect the binding efficiency of the predicted microRNA with hsa_circ_0007456, we designed relevant probes to pull down the predicted microRNA and examined the binding levels of hsa_circ_0007456 by RT-qPCR (Fig. 3A, B). We found that binding efficiency of miR-6852-3p with hsa_circ_0007456 was the highest among all the predicted microRNA (Fig. 3C). We further designed the biotin labeled hsa_circ_0007456 probe to detect the interaction in SMMC-7721 and QGY-7703 cell lines (Fig. 3D–G). As expected, the hsa_circ_0007456 probe enriched more hsa_circ_0007456 (*p* < 0.01, Fig. 3D, E), as well as miR-6852-3p (*p* < 0.01, Fig. 3F, G), than the negative control probe. Consistently, the biotin labeled miR-6852-3p enriched more hsa_circ_0007456 than the negative control probe (*p* < 0.01, Fig. 3H, I).

Through sequence analysis, we found that hsa_circ_0007456 indeed shares miRNA response elements (MREs) of miR-6852-3p (Fig. 3J). To further explore the function of this interaction, the luciferase plasmids containing the wild-type 3′ terminal (WT) or the MRE deleting 3′ terminal (MUT) of hsa_circ_0007456 were constructed (Fig. 3J) and simultaneously co-transfected with miR-6852-3p mimic, negative control or inhibitor into SMMC-7721 (Fig. 3K) or QGY-7703 (Fig. 3L). After that, the luciferase activity of each combination was detected. It showed that the miR-6852-3p mimic significantly suppressed the luciferase activity of WT (*p* < 0.01, Fig. 3K, L, Column 2) but not MUT (Fig. 3K, L, Column 5). Conversely, the miR-6852-3p inhibitor significantly increased the luciferase activity of WT (*p* < 0.01, Fig. 3K, L, Column 3) but not MUT (Fig. 3K, L, Column 6), indicating that hsa_circ_0007456 acts as a sponge of miR-6852-3p.

In addition, we examined the expression levels of miR-6852-3p in the primary HCC tissues (Fig. 3M) and portal vein tumor thrombus (Fig. 3N), and made the correlation analysis with the expression levels of hsa_circ_0007456. We found that the expression levels of miR-6852-3p were negatively correlated with the expression levels of hsa_circ_0007456, suggesting that hsa_circ_0007456 may function as a negative regulator of miR-6852-3p.

### The expression levels of miR-6852-3p could also regulate the susceptibility of HCC cell lines to NK cells

**Fig. 4:**
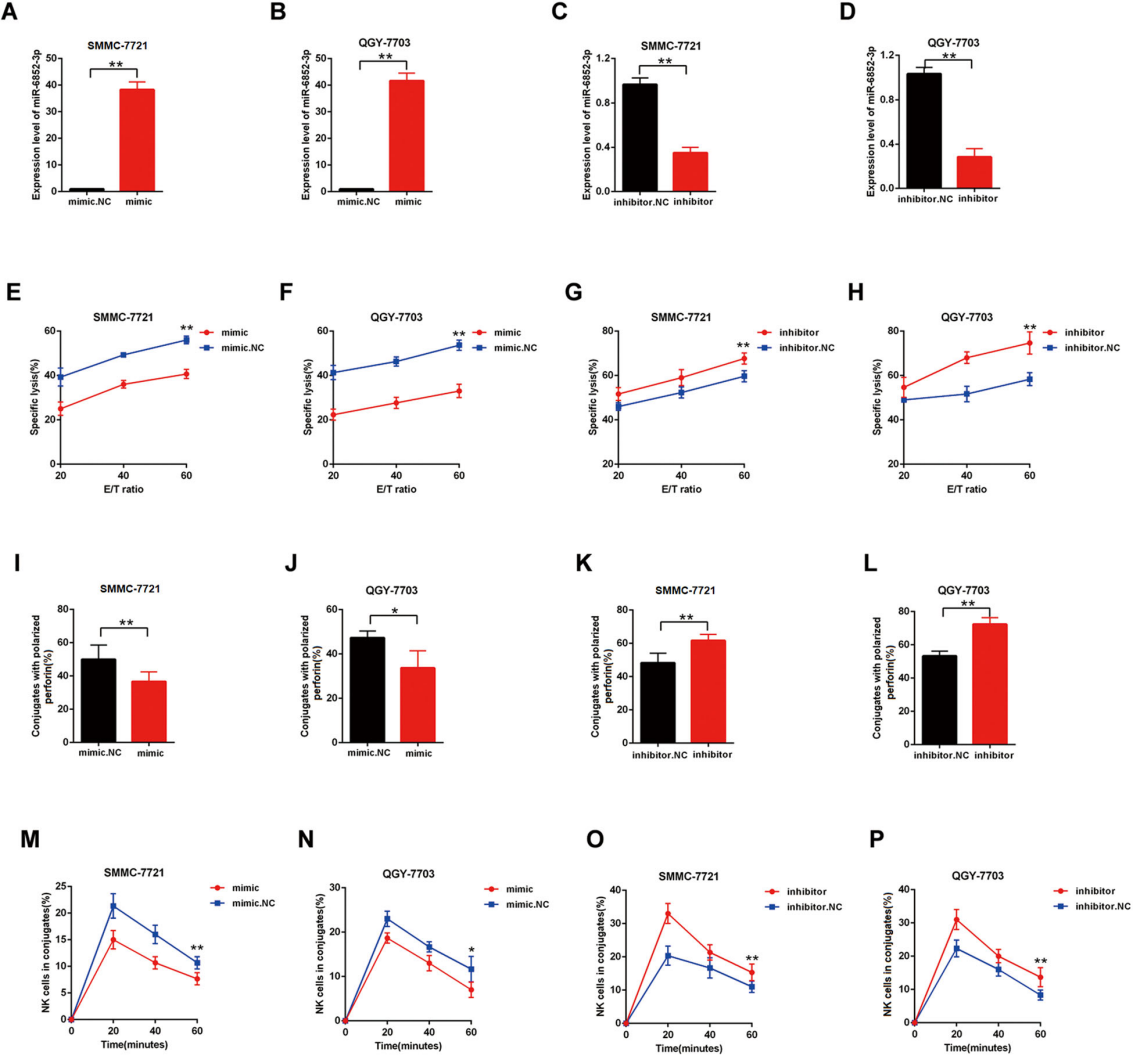
The upregulation of miR-6852-3p decreased the susceptibility of HCC to NK cells. After transfection with mimic miR-6852-3p or inhibitor in SMMCC-7721 or QGY-7703: **A**–**D** The relative expression levels of miR-6852-3p were detected by RT-qPCR; **E**–**H** The NK cell cytotoxicity to the transfected cells was assessed by calcein release assays in indicated E/T ratios; (I-L) The numbers of perforin-containing NK cells against to the transfected cells were counted and analyzed; (M-P) The conjugate formation between NK cells with the transfected cells was assessed by conjugation assay. Data are representative of three independent experiments and are presented as mean ± SD. *p < 0.05; **p < 0.01.

To explore the role of miR-6852-3p in the NK resistance of HCC cells, we respectively transfected SMMC-7721 and QGY-7703 with miR-6852-3p mimic (Fig. 4A, B) or inhibitor (Fig. 4C, D), and examined their susceptibility to NK cells through the calcein release assay (Fig. 4E–G), perforin polarization assay (Fig. 4I–L), and conjugation assay (Fig. 4M–P). We found that the cells transfected with mimic were less susceptible than the control cells (Fig. 4E, F, I, J, M, N), while the cells transfected with inhibitor were more susceptible than the control cells (Fig. 4G, H, K, L, O, P). These results suggest that the expression levels of miR-6852-3p could also regulate the susceptibility of HCC cell lines to NK cells.

### ICAM-1 was validated as the functional target of miR-6852-3p in HCC

**Fig. 5:**
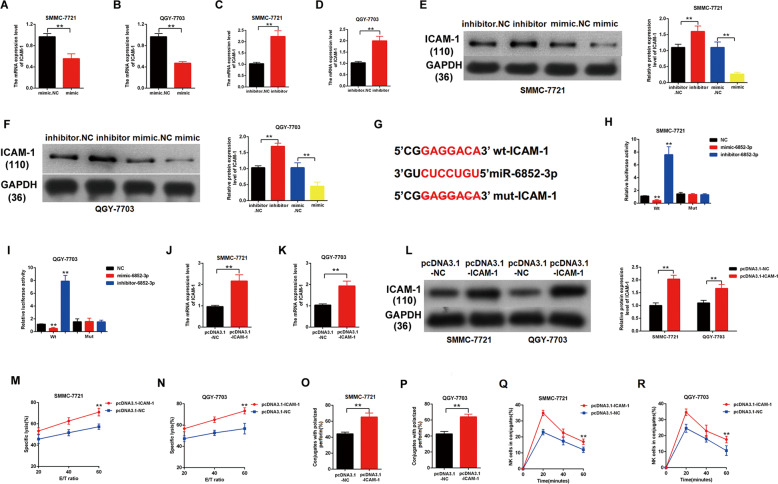
ICAM-1 is the functional target of miR-6852-3p. **A**–**F** After transfection with mimic miR-6852-3p or negative control in SMMCC-7721 or QGY-7703, the relative RNA (**A**–**D**) or protein (**E**, **F**) levels of ICAM-1 were detected by RT-qPCR or western blotting. **G** The predicted binding motif between ICAM-1 and miR-6852-3p is indicated in red, and the mutant ICAM-1 was designed without the complementary sequence. **H**, **I** SMMCC-7721 or QGY-7703 stably expressing the luciferase construct containing the wild-type (WT) or mutant (MT) ICAM-1 were respectively transfected with miR-6852-3p mimic, inhibitor or the negative control (NC), and then the luciferase activity was examined. **J**–**L** After transfection with pcDNA3.1-ICAM-1 or the vector control in SMMCC-7721 or QGY-7703, the relative RNA (**J**, **K**) or protein (**L**) levels of ICAM-1 were detected by RT-qPCR or western blotting. **M**, **N** The NK cell cytotoxicity to the ICAM-1-overexpressing cells or the control cells was assessed by calcein release assays in indicated E/T ratios. **O**, **P** The numbers of perforin-containing NK cells against the ICAM-1-overexpressing cells or the control cells were counted and analyzed. **Q**, **R** The conjugate formation between NK cells with the ICAM-1-overexpressing cells or the control cells was assessed by conjugation assay. Data are representative of three independent experiments and are presented as mean ± SD. **p < 0.01.

To find out the downstream target of miR-6852-3p, we used the miRBase for screening (http://www.mirbase.org/). As standard, the potential gene target should harbor a similar MRE with the seed sequence of miR-6852-3p and hsa_circ_0007456. Accordingly, we finally focused on ICAM-1, which has been reported to involve in target cell cytolysis induced by NK cells. To confirm this, we transfected SMMC-7721 and QGY-7703 with miR-6852-3p mimic or inhibitor (Fig. 5A–D), and examined the expression of ICAM-1 by western blotting (Fig. 5E, F). As expected, the transfection of mimic resulted in an obvious decrease in the protein levels of ICAM-1, while the transfection of inhibitor resulted in an obvious increase in the protein levels of ICAM-1 (Fig. 5E, F).

Next, we constructed the luciferase plasmids containing the wild-type MRE (WT) or the MRE deleting mutant (MUT) of ICAM-1 (Fig. 5G). Either of these two plasmids was co-transfected with miR-6852-3p mimic, negative control or inhibitor in SMMC-7721 or QGY-7703, and then the luciferase activity was examined. We found that the miR-6852-3p mimic significantly suppressed the luciferase activity of the WT (*p* < 0.01, Fig. 5H, I, Column 2) but not the MUT (Fig. 5H, I, Column 5). In contrast, the miR-6852-3p inhibitor significantly increased the luciferase activity of the WT (*p* < 0.01, Fig. 5H, I, Column 3) but not the MUT (Fig. 5H, I, Column 6).

To further study the role of ICAM-1 in the NK resistance of HCC cells, we transfected SMMC-7721 and QGY-7703 with pcDNA3.1-ICAM-1 plasmid (Fig. 5J–L), and examined their susceptibility to NK cells through the calcein release assay (Fig. 5M, N), perforin polarization assay (Fig. 5O, P) and conjugation assay (Fig. 5Q, R). We found that the upregulation of ICAM-1 made the tumor cells more susceptible to NK cells (Fig. 5M–R), suggesting that ICAM-1 is the functional target of miR-6852-3p in HCC.

### The hsa_circ_0007456 regulated the expression of ICAM-1 by sponging miR-6852-3p

**Fig. 6:**
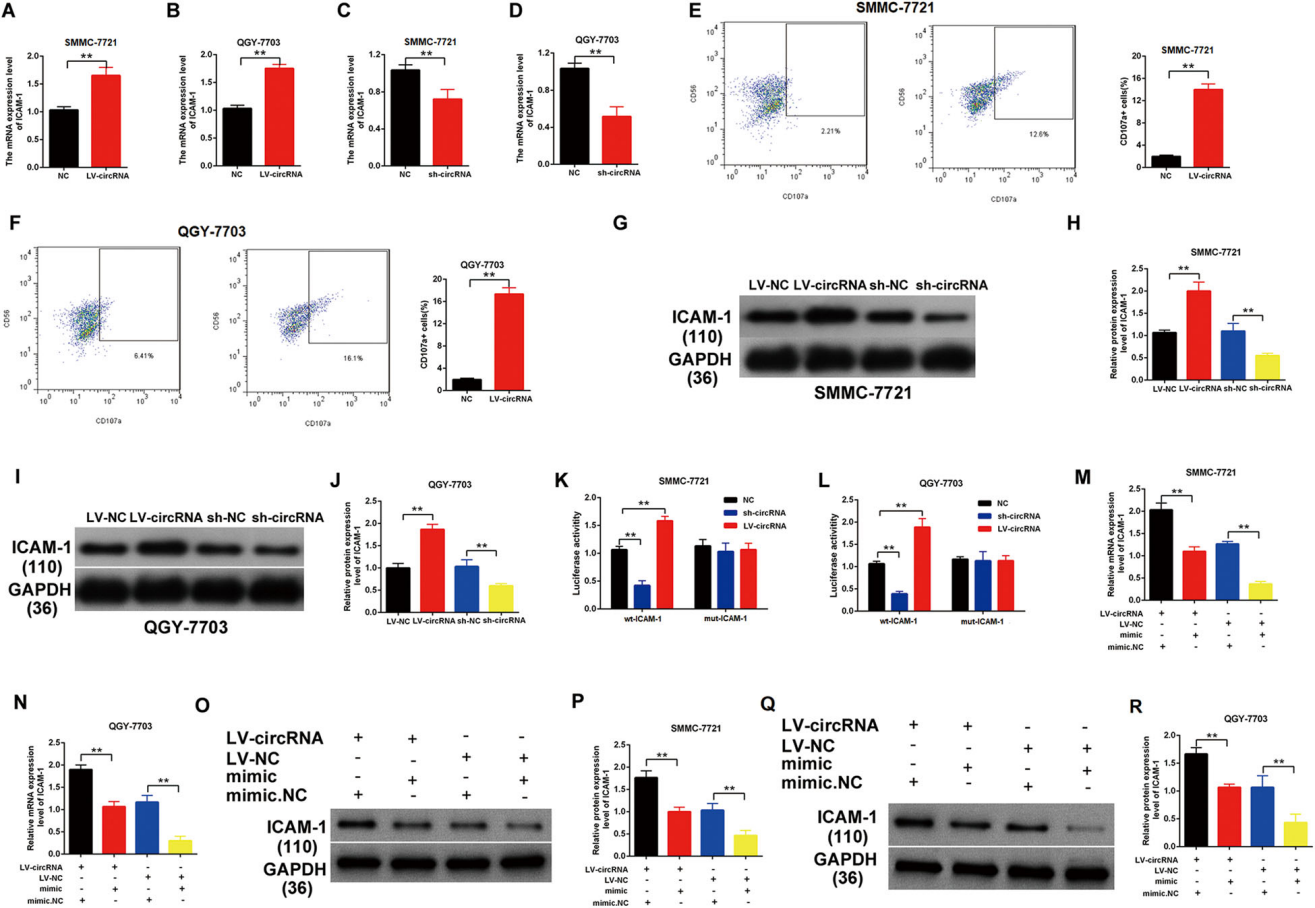
The hsa_circ_0007456 regulated the expression of ICAM-1 by sponging miR-6852-3p. **A**–**J** After infection with hsa_circ_0007456-overexpressing or shRNA lentivirus or the control lentivirus in SMMCC-7721 or QGY-7703, the relative RNA levels of ICAM-1 were detected by RT-qPCR (**A**–**D**); the expression of ICAM-1 (CD107a) on the cell surface was detected by flow cytometry (**E**, **F**); and the relative protein levels of ICAM-1 were detected by western blotting (**G**–**J**). **K**, **L** SMMCC-7721 or QGY-7703 stably expressing the luciferase construct containing the wild-type (WT) or mutant (MT) ICAM-1 were respectively infected with hsa_circ_0007456-overexpressing or shRNA lentivirus or the negative control lentivirus (NC), and then the luciferase activity was examined. **M**–**R** After treatment with the indicated hsa_circ_0007456 or the control lentivirus plus miR-6852-3p mimic or negative control (NC) in SMMCC-7721 or QGY-7703, the mRNA levels (**M**, **N**) and the protein levels (**O**–**R**) of ICAM-1 were respectively detected by RT-qPCR and western blotting. Data are representative of three independent experiments and are presented as mean ± SD. **p < 0.01.

To confirm the hsa_circ_0007456/miR-6852-3p/ICAM-1 axis, we further examined the mRNA (Fig. 6A–D) or protein levels (Fig. 6E–J) of ICAM-1 in the hsa_circ_0007456-overexpressing cell lines and hsa_circ_0007456-downregulated cell lines. We found that the overexpression of hsa_circ_0007456 significantly enhanced the expression of ICAM-1 (Fig. 6A, B, E, F, G–J), but when the expression of hsa_circ_0007456 was downregulated by shRNA, the expression of ICAM-1 decreased as well (Fig. 6G–J).

Next, we transfected the luciferase plasmids containing the wild-type MRE (WT) or the MRE deleting mutant (MUT) of ICAM-1 into the hsa_circ_0007456-overexpressing cell lines and hsa_circ_0007456-downregulated cell lines (Fig. 6K, L). We found that the overexpression of hsa_circ_0007456 significantly enhanced the luciferase activity of the WT (*p* < 0.01, Fig. 6K, L, Column 2) but not the MUT (Fig. 6K, L, Column 5), while the downregulation of hsa_circ_0007456 significantly suppressed the luciferase activity of the WT (*p* < 0.01, Fig. 6K, L, Column 3) but not the MUT (Fig. 6K, L, Column 6).

In addition, we transfected the miR-6852-3p mimic into the cell lines with or without the overexpression of hsa_circ_0007456, and then examined the expression of ICAM-1. As expected, the overexpression of hsa_circ_0007456 significantly enhanced the expression of ICAM-1, but the transfection of mimic significantly suppressed and partially rescued the expression of ICAM-1 (*p* < 0.01, Fig. 6M–R), indicating that hsa_circ_0007456 and miR-6852-3p counteract with each other in the regulation of ICAM-1.

### The miR-6852-3p/ICAM-1 axis is essential for the NK cytotoxicity toward HCC mediated by hsa_circ_0007456

**Fig. 7:**
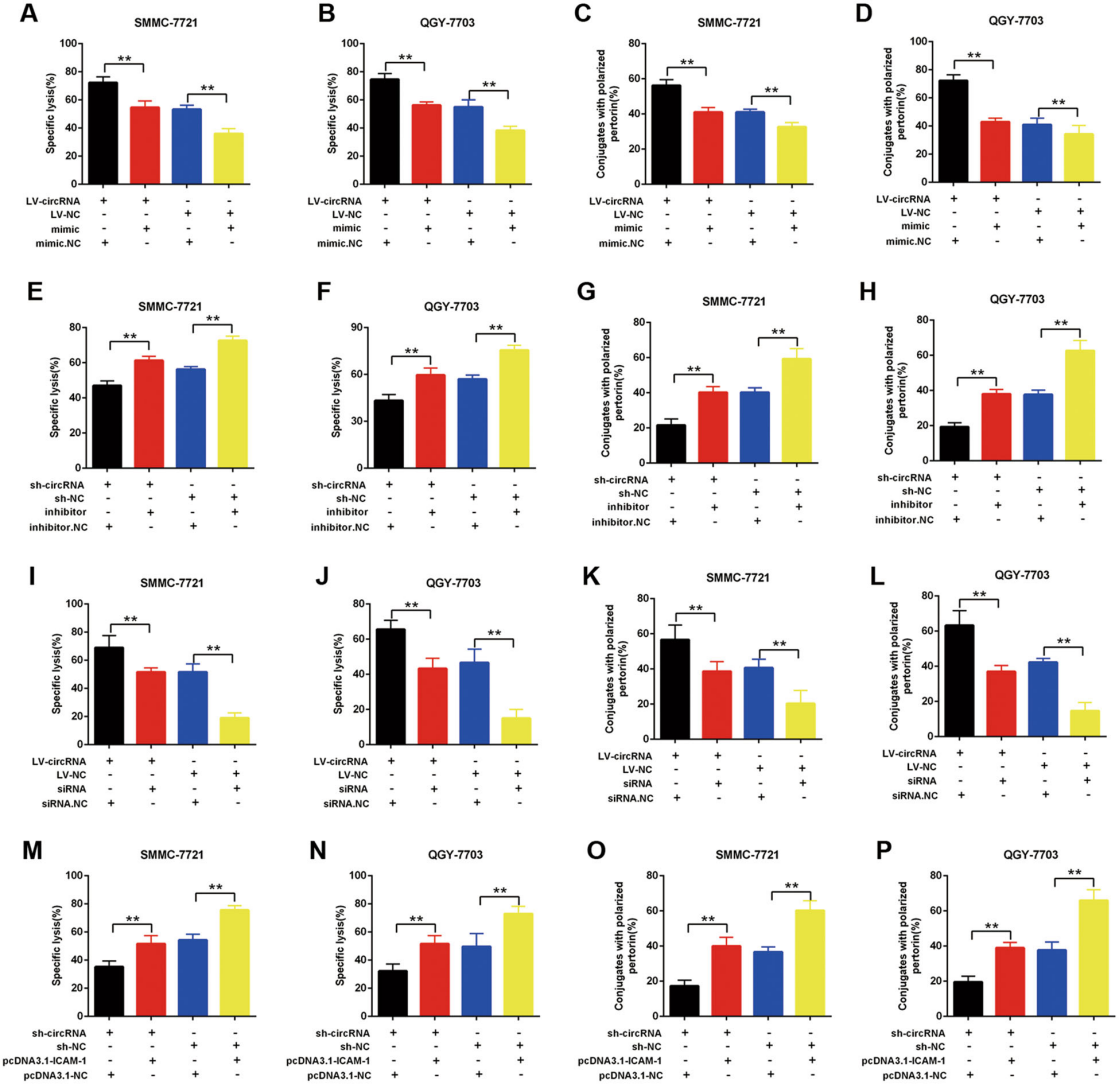
The hsa_circ_0007456 regulated the susceptibility of HCC to NK cells by targeting miR-6852-3p/ICAM-1 axis. **A**–**D** After treatment with the indicated hsa_circ_0007456-overexpressing or the control lentivirus plus miR-6852-3p mimic or negative control (NC) in SMMCC-7721 or QGY-7703, the NK cell cytotoxicity to the transfected cells was assessed by calcein release assays in indicated E/T ratios (**A**, **B**), while the numbers of perforin-containing NK cells against to the transfected cells were counted and analyzed (**C**, **D**). **E**–**H** After treatment with the indicated hsa_circ_0007456-shRNA or the control lentivirus plus miR-6852-3p inhibitor or negative control (NC) in SMMCC-7721 or QGY-7703, the NK cell cytotoxicity to the transfected cells was assessed by calcein release assays in indicated E/T ratios (**E**, **F**), while the numbers of perforin-containing NK cells against to the transfected cells were counted and analyzed (**G**, **H**). **I**–**L** After treatment with the indicated hsa_circ_0007456-overexpressing or the control lentivirus plus ICAM-1 siRNA or negative control (NC) in SMMCC-7721 or QGY-7703, the NK cell cytotoxicity to the transfected cells was assessed by calcein release assays in indicated E/T ratios (**I**, **J**), while the numbers of perforin-containing NK cells against to the transfected cells were counted and analyzed (**K**, **L**). **M**–**P** After treatment with the indicated hsa_circ_0007456-shRNA or the control lentivirus plus pcDNA3.1-ICAM-1 or negative control (NC) in SMMCC-7721 or QGY-7703, the NK cell cytotoxicity to the transfected cells was assessed by calcein release assays in indicated E/T ratios (**M**, **N**), while the numbers of perforin-containing NK cells against to the transfected cells were counted and analyzed (**O**, **P**). Data are representative of three independent experiments and are presented as mean ± SD. **p < 0.01.

To further explore the role of miR-6852-3p/ICAM-1 axis in the NK cytotoxicity toward HCC mediated by hsa_circ_0007456, we transfected the miR-6852-3p mimic into the hsa_circ_0007456-overexpressing SMMC-7721 and QGY-7703 cells, and examined the their susceptibility to NK cells through the calcein release assay (Fig. 7A, B) and conjugation assay (Fig. 7C, D). As expected, the overexpression of hsa_circ_0007456 made the HCC cells more sensitive to NK cytolysis (Fig. 7A–D), but the transfection of mimic partially rescued this phenotype (*p* < 0.01, Fig. 7A–D, Column 2 vs. Column 1). Similarly, the transfection of miR-6852-3p inhibitor significantly counteracted the NK cytolysis inhibiting effect induced by hsa_circ_0007456 shRNA (*p* < 0.01, Fig. 7E–H, Column 2 vs. Column 1).

In addition, we transfected ICAM-1 siRNA into the hsa_circ_0007456-overexpressing SMMC-7721 and QGY-7703 cells, and found that the downregulation of ICAM-1 significantly counteracted the NK cytolysis promoting effect induced by the overexpression of hsa_circ_0007456 (*p* < 0.01, Fig. 7I–L, Column 2 vs. Column 1). Similarly, the overexpression of ICAM-1 significantly counteracted the NK cytolysis inhibiting effect induced by hsa_circ_0007456 shRNA (*p* < 0.01, Fig. 7M–P, Column 2 vs. Column 1). These results demonstrate that the miR-6852-3p/ICAM-1 axis is essential for the NK cytotoxicity toward HCC mediated by hsa_circ_0007456.

## Discussion

There are numbers of studies implying that circular RNA may participate in the progression of diseases including cancer, diabetes etc^15,16^. In this study, we found that hsa_circ_0007456 was significantly downregulated in HCC tumor tissues compared with normal liver tissues, and that the lower expression of hsa_circ_0007456 is associated with the poorer progression of HCC (Fig. 1). In a previous study, hsa_circ_0007456 related network was also reported to be highly correlated with the pathogenesis of HCC^17^. Combined with our analysis, we hypothesize that the downregulation of hsa_circ_0007456 may be an important step for the progression of HCC.

Within tumor microenvironment, NK cells are an important immune cell type for tumor immunosurveillance, and the NK cell immunodeficiency is significantly associated with the occurrence of malignancies^18^. However, it’s still unclear how cancer cells regulate themselves to escape the surveillance of NK cells. In this study, we found that the susceptibility of the tumor cells to NK cells was significantly regulated in response to the level of hsa_circ_0007456. The lower expression of hsa_circ_0007456 was associated with less conjugation in NK cells (Fig. 2), suggesting that hsa_circ_0007456 could mediate the interaction between HCC cancer cells and NK cells.

It has been known that circRNAs could interact with micoRNA through the miRNA response elements (MREs)^10^. Through a series of screening and validation, we further revealed that the main downstream target of hsa_circ_0007456 is miR-6852-3p, which can directly target and downregulate the expression of ICAM-1 (Figs. 3–5). Functionally, hsa_circ_0007456 and miR-6852-3p counteract with each other to regulate the susceptibility of HCC to NK cells (Figs. 6 and 7). In this study, we also found that ICAM-1 is the downstream target of miR-6852-3p. It has been previously reported that the expression of ICAM-1 on human cancer cells could regulate the adhesion between NK cells and target cells through its binding with lymphocyte function–associated antigen 1 (LFA-1) on NK cells^19,20^. Therefore, we believed that ICAM-1 should be an essential checkpoint in the downstream of hsa_circ_0007456 in the regulation of the interaction between HCC tumor cells and NK cells.

In conclusion, we identify here hsa_circ_0007456 as a promising biomarker of HCC. We also highlight hsa_circ_0007456/miR-6852-3p/ICAM-1 axis as an important signaling pathway in the process of tumor immune evasion and the tumorigenesis of HCC.

## Materials and methods

### Patients and patient samples

Liver cancer tissues, the matched adjacent normal tissues and the matched regional lymph nodes were collected from 72 patients treated with liver resection at Taizhou Central Hospital (Taizhou University Hospital) between April 2015 and August 2017. The information of age and gender of each patient was recorded in detail. The evaluation of the primary tumor stage, the assessment of lymph node metastasis, the detection of HBV infection and the genetic testing of the samples were performed in the pathology department of Taizhou Central Hospital (Taizhou University Hospital). All the cases were staged according to the TNM staging system of the AJCC 8th edition. The collected tissues were stored at −80 °C. Our current study was approved by the Ethics Committee of Taizhou Central Hospital (Taizhou University Hospital). All the patients provided thee written informed consent before the study.

### Cell culture and transfection

The HCC cell lines, including SMMC-7721, QGY-7703, Hep3B, Huh-7, HepG2, and the human hepatic cell line L-02 were purchased from the Cell Bank of Chinese Academy of Sciences. L-02 was cultured in RPMI-1640 medium supplemented with 20% FBS. SMMC-7721 and QGY-7703 were cultured in RPMI-1640 medium supplemented with 10% FBS. Hep3B and HepG2 were cultured in MEM medium supplemented with 10% FBS. Huh-7 was cultured in DMEM medium supplemented with 10% FBS. All the cell lines were maintained in the incubator with 5% CO_2_ at 37 °C, and were tested for mycoplasma contamination during culturing.

### Plasmids, microRNA, and siRNA

The information of hsa_circ_0007456 was obtained from CircBase (http://circbase.org, genome position: chr17: 11984672-12016677) and was annotated by CircPrimer software (http://www.bioinf.com.cn/). The cDNA of hsa_circ_0007456 was cloned into pLO-ciR vector. The target sequence of shRNA against hsa_circ_0007456 is 5′-GGCCATACATGGCAAGAGAGA-3′ and was cloned into pLKO.1 vector. The sequence of ICAM-1 was obtained from NCBI (NM_001319035) and was cloned into pcDNA3.1(+) vector. The synthesized microRNA mimic, inhibitor, siRNA and the negative control were purchased from RIBOBIO.

### Biotin labeled probe pull down assay

The biotin labeled hsa_circ_0007456 or microRNA probes were synthesized by RIBOBIO. For purification, ~1 × 10^7^ cells were collected and lysed in lysis buffer. After that, 3 μg biotin labeled probe was added to the buffer and incubated at room temperature for 4 h. To pull down the circRNA-microRNA complex, strepavidin magnetic beads (Thermo Fisher, #88816) were added to the buffer and slowly rotated for another 4 h, followed by washing step for four times. Finally, the beads were collected by centrifuge, and the binding RNA was extracted with TRIzol reagent. Each group of the pull down assay was performed in triplicate, and was repeated independently for three times.

### Reverse transcription and quantitative PCR (RT-qPCR)

The RT-qPCR experiments were performed according the manufacture protocol (miScript II RT Kit, QIAGEN) on the ABI 7500 Real-Time PCR System. Each sample was examined in triplicate from three independent experiments. The sequence of RT primer for miR-6852-3p is 5′-GTCGTATCCAGTGCAGGGTCCGAGGTATTCGCACTGGATACGACCATGTC-3′. The qPCR primers used in this study are as follows:

**Table:** 

hsa_circ_0007456	(Forward: 5′-GCTCTGTGACTTCGGCATCAG-3′;
	Reverse: 5′-CAGAACCATAAGCTCCTCGTCC-3′),
miR-6852-3p	(Forward: 5′-CCCTGGGGTTCTGAGGACATG-3′;
	Reverse: 5′-GTGCAGGGTCCGAGGT-3′),
U6	(Forward: 5′-GCTTCGGCAGCACATATACTAAAAT-3′;
	Reverse: 5′-CGCTTCACGAATTTGCGTGTCAT-3′),
ICAM-1	(Forward: 5′-CCGTGTACTGGACTCCAGAACG-3′;
	Reverse: 5′-GGCTCCATGGTGATCTCTCCTC-3′),
β-Actin	(Forward: 5′-CCACCCTCTCTAGTCTAAAGAGC-3′;
	Reverse:5′-CTCCTTAATGTCACGCACGAT-3′).

The levels of hsa_circ_0007456 and miR-6852-3p were normalized to U6 small nuclear RNA levels. The levels of ICAM-1 were normalized to β-Actin RNA levels. Each RT-qPCR experiment was performed independently for at least twice.

### Western blotting

We collected the same number of cells from each group. Cells were extracted with 1% SDS cell lysis/loading buffer. All the protein samples were subjected to electrophoresis by SDS-PAGE method and then transferred to PVDF membrane for further immunoblot. The primary antibodies used in this study include: ICAM-1 antibody (Proteintech, #10831-1-AP, 1:1000), GAPDH antibody (Proteintch, #60004-1-Ig, 1:1000). The blots were exposed using chemiluminescence and photographed by Tannon 3500 Imager. Photos were analyzed by ImageJ software. Each western blotting experiment was performed independently for at least twice.

### Luciferase reporter assay

The hsa_circ_0007456 3′ terminal fragment (200 bp) or ICAM-1 3′UTR was inserted into the 3′ terminal of the luciferase gene in pGL3 vector (pGL3-3′) and sequenced before use. About 1 × 10^5^ cells were seeded in 48-well plate before transfection. After attachment, the cells of each well were respectively transfected with 320 ng of pGL3-3′ and 30 ng of pRL-TK containing Renilla luciferase plus miR-6852-3p mimic, mimic negative control or inhibitor (RIBOBIO) using Lipfatamin 2000 (Invitrogen, #11668019). In total, 48 h after transfection, the relative luciferase absorbance value of each group was examined by Dual-Luciferase Reporter Assay System (Promega, #E1910) and normalized to Renilla luciferase absorbance value. Each group of the luciferase reporter assay was performed in triplicate, and was repeated independently for three times.

### NK cell purification and expansion

The purification and expansion of human NK cells was referred to the previous study. Briefly, human peripheral blood mononuclear cells (PBMC) were acquired from healthy donors, and then were incubated with K562 aAPC (antigen-presenting cell) receiving 100 Gy radiation with 1:2 ratio (PBMC: K562 aAPC). The expanded NK cells used in this study were cultured for 14 to 21 days.

### Calcein release assay

In this assay, we used 30 μM calcein-AM (Dojindo) to stain the target cells at 37 °C for 30 min. Then the stained target cells were mixed with NK cells at a series of E/T ratios (20, 40 and 60) respectively. The mixed cells were then seeded in 96-well plates. After culture for 3 h, 100 μl of supernatant from each well was harvested for fluorescent detection (excitation filter: 490 nm). The supernatant from the well cultured with target cells alone was tested as spontaneous release value, while the supernatant from the well treated with 2% Triton x-100-containing medium was tested as maximum release value. The specific lysis was calculated by [(test release value – spontaneous release value)/(maximum release value-spontaneous release value)]. Each group of the calcein release assay was performed in triplicate, and was repeated independently for three times.

### Perforin polarization assay

Firstly, the target cells and the NK cells were mixed with 1:1 ratio and incubated at 37 °C for 30 min. Then, the mixed cells were transferred to 12-well plates placed with poly-D-lysine-coated slides, and were incubated at 37 °C for 1 h. After that, the slides were harvested, fixed in 4% paraformaldehyde, permeablized with 0.5% Triton X-100 in PBS, and stained with anti-Perforin antibody and with Alexa Fluor 568-conjugated secondary antibody. Lastly, the slides were observed and photographed with confocal microscope. The polarization was scored for NK cells in contact with the target cells. Each group of the perforin polarization assay was performed in triplicate, and was repeated independently for three times.

### Conjugation assay

In this assay, we also used 30 μM calcein-AM (Dojindo) to stain the target cells at 37 °C for 30 min. Then the stained target cells were mixed with NK cells with 1:2 ratio. The mixed cells were incubated at 37 °C for 0, 20, 40, 60 min and then harvested for flow cytometry analysis. The events with calcein and perforin double-positive were consider as conjugated cells. The ratio of the NK cells in conjugation was calculated by [the double-positive events]/ [the total events]. Each group of the conjugation assay was performed in triplicate, and was repeated independently for three times.

### Statistical analysis

Regression analysis was performed with Graphpad software. Data statistical analysis was performed with SPSS software or SAS software. To campare the expression levels of hsa_circ_0007456 between the HCC samples from patients with different clinical pathological characteristics, student t test was used (Fig. 1). To analyze the difference of the distribution of high expression or low expression of hsa_circ_0007456 in the HCC patients with different clinical pathological characteristics, chi-square test was used (Table 1). For the functional experiments in cell lines, Student’s *t* test was used to compare the difference of data between groups. Data are presented as mean ± SD. *p* < 0.05 represents significant difference. *p < 0.05, **p < 0.01 and ***p < 0.001, respectively.

## Supplementary information

Supplementary Table 1

Supplementary Table 2

## Data Availability

All data are available upon request.
